# Spinal muscular atrophy patient-derived motor neurons exhibit hyperexcitability

**DOI:** 10.1038/srep12189

**Published:** 2015-07-20

**Authors:** Huisheng Liu, Jianfeng Lu, Hong Chen, Zhongwei Du, Xue-Jun Li, Su-Chun Zhang

**Affiliations:** 1 Waisman center, University of Wisconsin, Madison, WI, 53705, USA; 2 Department of Neuroscience and Department of Neurology, School of Medicine and Public Health, University of Wisconsin, Madison, WI 53705, USA; 3 Department of Neuroscience, University of Connecticut Health Center, Farmington, CT 06030, USA

## Abstract

Spinal muscular atrophy (SMA) presents severe muscle weakness with limited motor neuron (MN) loss at an early stage, suggesting potential functional alterations in MNs that contribute to SMA symptom presentation. Using SMA induced pluripotent stem cells (iPSCs), we found that SMA MNs displayed hyperexcitability with increased membrane input resistance, hyperpolarized threshold, and larger action potential amplitude, which was mimicked by knocking down full length survival motor neuron (SMN) in non-SMA MNs. We further discovered that SMA MNs exhibit enhanced sodium channel activities with increased current amplitude and facilitated recovery, which was corrected by restoration of SMN1 in SMA MNs. Together we propose that SMN reduction results in MN hyperexcitability and impaired neurotransmission, the latter of which exacerbate each other via a feedback loop, thus contributing to severe symptoms at an early stage of SMA.

Spinal muscular atrophy (SMA) is one of the most common genetic causes of infant mortality. It is caused by loss or mutation of survival motor neuron 1 (*SMN1*) gene and is characterized by degeneration of spinal cord motor neurons (MN) and severe muscle weakness/atrophy^1^,^2^,^3^. There is presently no effective treatment for SMA.

Human *SMN* has two genes, *SMN1* and *SMN2*, whereas most animals have only *SMN1* gene. SMN1gene primarily produces the full length SMN (SMN-FL) protein whereas SMN2 gene mostly (80–90%) translates to a truncated, unstable protein lacking exon 7 (SMNΔ7) and only 10% full length proteins^4^,^5^,^6^,^7^. Hence, the onset and severity of SMA is largely dependent on the *SMN2* copy numbers. This is mimicked by transgenic expression of *SMN2* in animals with deletion of the endogenous *SMN1*^8^,^9^. Interestingly, SMA transgenic mice often show limited motor neuron degeneration at an early stage despite severe phenotypes, including muscle weakness and atrophy. For examples, severe SMA mice show difficulty in movement, suckling and breathing by postnatal day 2 (P2) before significant MN loss after P3^9^. SMA-Δ7 mice exhibit early impairments of motor behavior and die at approximately 2 weeks, but MN loss is not detectable before P4 and is modest even at end-stage of P13^10^,^11^,^12^. Mild SMA mice, SMN (A2G), begin to display signs of muscle weakness at 3 week of age, whereas MN loss occurs at ~3 months^13^. This dichotomy suggests that most SMA phenotypes are likely attributed to functional impairment rather than physical loss of MNs. Indeed, abnormal neuronal activity, hyperexcitability with hyperpolarized threshold and enlarged action potential (AP), has recently been reported in MNs at P3-4 from SMA-∆7 mice^14^ and severe SMA mice^15^. However, the cause-effect relationship between SMN and neuronal hyperexcitability is not firmly established. Importantly, how SMN mutation results in neuronal hyperexcitability is not known.

Induced pluripotent stem cells (iPSCs) from patients with neurological conditions offer an opportunity to reveal early pathological changes directly in patient neurons^16^,^17^,^18^,^19^,^20^. Analysis of SMA iPSCs^21^,^22^,^23^,^24^ or SMN knockdown hPSCs^25^ indicates a loss of MNs at varied degrees, consistent with the degenerative nature of SMA. MNs from SMA iPSCs or SMN knockdown hPSCs display delayed neurite outgrowth^21^,^25^ and increased apoptosis^24^. Nevertheless, early events that ultimately lead MN degeneration, including functional alteration, remain unknown.

We have established a system to generate enriched functional MNs^26^ which enables identification of early disease phenotypes^27^. Using a similar system from three type-I SMA patients and three non-SMA individuals, we discovered that SMA MNs exhibited hyperexcitability with hyperpolarized threshold and larger AP amplitude. This phenotype is corrected by expression of SMN and mimicked in normal MNs by knocking down of SMN. Further analysis revealed enhanced Na^+^ -channel activities in SMA MNs as compared to normal MNs.

## Results

### MN generation from SMA iPSCs is not altered

We generated iPSCs from three type-1 SMA patients (SMA-1, SMA-2 and SMA-3 from GM03813, GM09677, and GM00232 fibroblasts, respectively) and two controls from two carrier individuals (Contl-2 and Contl-3 from GM03814 and GM03815 fibroblasts, respectively) using retrovirus^28^. In addition, we used a human ESC line, WA09 (also known as H9), as an extra control (Contl-1; Supplementary Table S1). GM03813 and GM09677 have 2 copies of SMN2 gene with homozygous deletion of exons 7 and 8 in SMN1 gene; GM00232 has one copy of the SMN2 gene with homozygous deletion of exons 7 and 8 in SMN1 gene (Supplementary Table S1). All iPSCs became stable cell lines (Supplementary Fig. S1), exhibited typical morphology with positive alkaline phosphatase staining, and expressed pluripotency markers including NANOG, SOX2, OCT4, SSEA-4 and TRA-1-60 (Supplementary Fig. S1a). They retained karyotype stability (Supplementary Fig. S1b), showed expression of endogenous, but not exogenous, pluripotent transcription factors (Supplementary Fig. S1c), and generated teratomas *in vivo* (Supplementary Fig. S1d).

**Figure 1:**
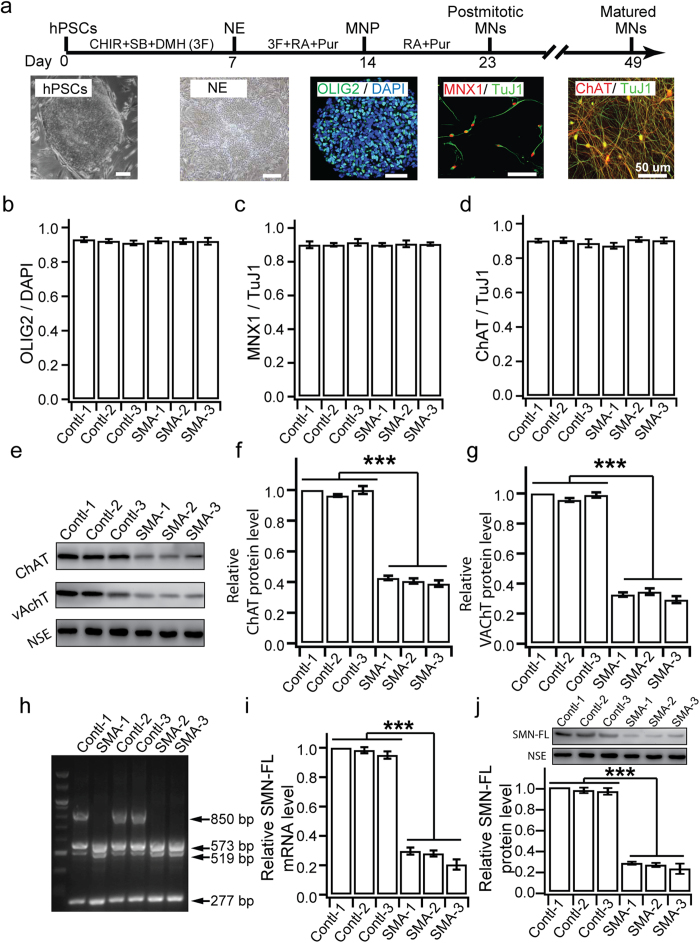
Differentiation and gene expression of MNs. (**a**) Schematic diagram of MN differentiation. Human PSCs were differentiated to neuroepithelia (NE) in the presence of 3 small molecules (3F: SB431542, ChIR99021 and DMH1) for 1 week, then to OLIG2^+^ motor neuron progenitors (MNP) with the addition of retinoic acid (RA) and purmorphamine (Pur) for 2 week, which were cultured in suspension for 1 week before being plated for differentiation to postmitotic MNX1^+^ MNs and ChAT^+^ MNs. (**b–d**) Quantification of the percentage of OLIG2^+^ MNPs at d14 (**b**), MNX1^+^ postmitotic MNs at 48-hour after plating (**c**, d23), and ChAT^+^ mature MNs at d49 (**d**). (**e**) Western blotting shows the expression of ChAT and VAchT at the 7^th^ week after differentiation. All the groups were collected and sampled under the same conditions. The cropped blots images are shown in the figure and the full-length blots are presented in Supplementary Fig. S3a. (**f–g**) Relative protein level of ChAT (**f**) and VAchT (**g**) in MNs for each group measured by western blots. (**h**) Dde I doesn’t digest *SMN1* (850bp), but cut *SMN2* and truncated exon 7 deleted *SMN* (Δ*SMN7*), into 573bp and 519bp, respectively, and 277bp. (**i**) Relative expression of SMN-FL mRNA in MNs as measured by qPCR. (**j**) Western blots showing relative SMN-FL protein levels in MNs for each group. All the groups were collected and sampled under the same conditions. The cropped blots images are shown in the figure and the full-length blots are presented in Supplementary Fig. S3a. All data are presented as mean ± SEM. ***p < 0.001; one-way ANOVA with post hoc test. N = 3 independent experiments.

We first asked if the differentiation and survival of MNs is altered by SMN mutation. Using our recently established protocol (Fig. 1a), we found that SMA iPSCs, as well as control PSCs, efficiently differentiated to enriched populations (~90%) of OLIG2^+^MN progenitors (MNP) at day 14 (d14) as measured in sections of MNP clusters (Fig. 1b, Supplementary Fig. S2a). At d21, the MNP clusters were dissociated and plated onto the laminin substrate in the presence of compound E, a NOTCH inhibitor to block progenitor proliferation. Quantification at d23 indicated that SMA iPSCs produced a similar population (~90%) of MNX1^+^ MNs among total βIII-tubulin^+^ (TuJ1^+^) neurons as control PSCs (Fig. 1c and Supplementary Fig. S2b).

At 4 weeks after plating MNPs (or 7 weeks after iPSC differentiation (d49)), we found a similar population of ChAT^+^ neurons among total TuJ1^+^ neurons between SMA and control (Fig. 1d and Supplementary Fig. S2c), suggesting that the MN differentiation is not altered in SMA. However, Western blotting revealed that ChAT (Fig. 1e,f, and Supplementary Fig. S3a) and VAchT (Fig. 1e,g), were significantly reduced in SMA MNs, suggesting that transmitter synthesis and release may be affected in SMA MNs. We performed DNA PCR and Dde I digestion to confirm the lack of SMN1 gene in SMA ChAT^+^ neurons (Fig. 1h). qPCR and western blot further validated ~80% reduction of SMN-FL mRNA (Fig. 1i) and protein (Fig. 1j and Supplementary Fig. S3a) level in diseased MNs, respectively. Therefore, our data suggest that the reduction of SMN-FL does not appear to affect the differentiation of MNs but may affect the neurotransmission. We didn’t find different SMN-FL expression between carrier groups, GM03814 and GM03815, and H9 group. This result is consistent with a previous report using patient-derived iPSCs^22^, suggesting that *SMN1* is either stabilized or its expression is up-regulated in iPSCs-derived MNs *in vitro*.

### SMA MNs display abnormal passive properties

**Figure 2:**
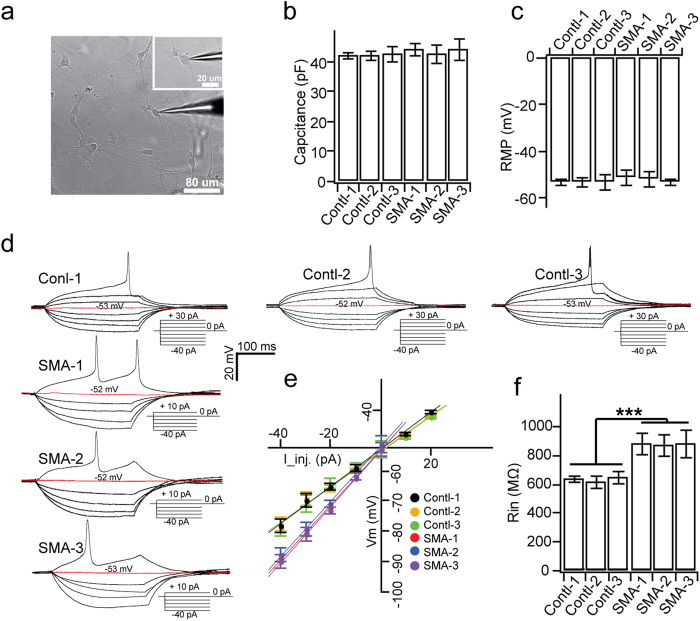
Passive membrane properties of SMA MNs. (**a**) A representative image of electrophysiological recording on individual MNs. Inset shows the enlarged image of the patched neuron. (**b,c**) Summary of average membrane capacitances (**b**) and resting membrane potentials (RMP, **c**) for each group. (**d**) Representative membrane voltage traces upon step injection of currents in control and SMA MNs. Inset: description of current injection from −40 pA to the injected current which induces the 1^st^ action potential. Red trace indicates the RMP at 0 pA injection. (**e**) Plot of membrane potential response (V_m_) that reached a steady-state against injected currents (I_inj.) in control and SMA MNs. Data points were fitted with a linear function to calculate the membrane input resistance (R_in_) by the slope. (**f**) Quantification of R_in_ from SMA and control MNs. All data shown represent mean ± SEM. ***p < 0.001; one-way ANOVA with post hoc test. N = 18 ~ 20 neurons for each group.

Human PSC-derived MNs using our current protocol usually become mature 2–4 weeks after plating MNPs, as indicated by firing APs and forming neuro-muscular junctions^26^,^27^. By whole-cell electrophysiological recording in SMA and control MNs (18–20 neurons / group) (Fig. 2a), we found that there was no difference between SMA and control MNs in membrane capacitance (Fig. 2b). Under current-clamp, we found that resting membrane potential (RMP) was similar between SMA and control MNs when currents were held at 0 pA (Fig. 2c). When currents (from –40 pA with 10 pA step for 500 ms) were injected into neurons until the first AP was triggered, we noted that 100% of recorded neurons from the SMA or control group fired APs, and membrane potential responses were enhanced in SMA as compared to control groups (Fig. 2d). By plotting peak membrane potential response upon current injection without AP occurrence (Fig. 2e), we found that membrane input resistance (R_in_) in SMA MNs was significantly larger than control MNs (Fig. 2f). The identity of the recorded cells (filled with neurobiotin during recording) was confirmed by immunostaining for ChAT (Supplementary Fig. S4). Therefore, human SMA MNs exhibit normal membrane capacitance and resting membrane potential, but increased membrane input resistance.

### SMA MNs exhibit hyperexcitability

**Figure 3:**
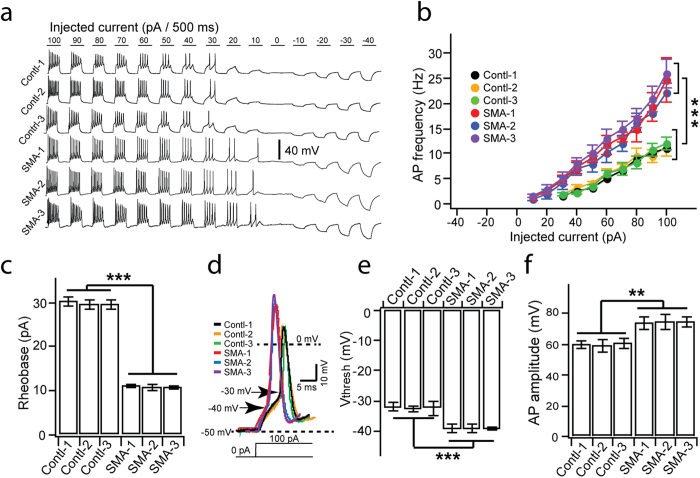
AP properties of SMA MNs. (**a**) Representative traces of induced APs upon current injections (from –40 pA to 100 pA with 10 pA step for 500 ms) in control and SMA MNs. (**b**) Plot of AP frequency against injected current size for each group (***p < 0.001; two-way ANOVA). (**c**) Summary of rheobase for control and SMA MNs (***p < 0.001; one-way ANOVA with post hoc test). (**d**) Representative individual AP induced by +100 pA injection in control and SMA MNs. Arrows indicate voltage threshold of AP. (**e** and **f**) Quantification of the voltage threshold (V_thresh_, **e**) and amplitude (**f**) of AP in different groups (**p < 0.01, ***p < 0.001; one-way ANOVA with post hoc test). All data shown represent mean ± SEM. N = 18 ~ 20 neurons for each group.

Increased input resistance under steady RMPs would suggest easy-going of AP. Indeed, upon current injection from −40 pA to +100 pA, AP frequency was significantly higher in SMA MNs than in control MNs at each given current injection (Fig. 3a,b). The minimal current required to trigger APs, rheobase, was +10 pA for SMA MNs as compared to +30 pA for control MNs (Fig. 3c). Detailed analysis of kinetics of individual APs induced by +100 pA current injection revealed that AP threshold (arrow in Fig. 3d) was significantly reduced in SMA MNs as compared to control MNs (Fig. 3e). Importantly, AP amplitude was significantly increased in SMA MNs (Fig. 3d, f). Thus, our data demonstrate that human SMA MNs are more readily excitable than control MNs, exhibiting hyperexcitability.

### Reduction of SMN-FL protein is responsible for hyperexcitability

**Figure 4:**
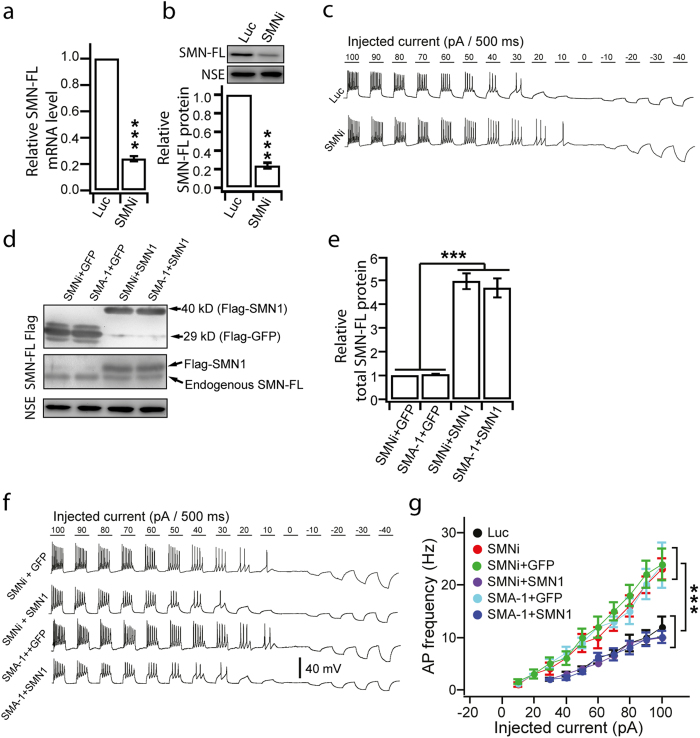
AP properties of control MNs after SMN-FL knockdown. (**a**) Relative expression of SMN-FL mRNA measured by qPCR in MNs differentiated from control (Luc) and SMN-FL knockdown (SMNi) hESCs group (***p < 0.001; unpaired Student’s t-test. N = 3 independent experiments). (**b**) Relative expression of SMN-FL proteins measured by western blots in MNs differentiated from Luc and SMNi group (***p < 0.001; unpaired Student’s t-test. N = 3 independent experiments). All the groups were collected and sampled under the same conditions. The cropped blots images are shown in the figure and the full-length blots are presented in Supplementary Fig. S3b. (**c**) Representative traces of induced APs upon current injections (from –40 pA to 100 pA with 10 pA step for 500 ms) in MNs differentiated from Luc and SMNi ESCs. (**d**) The cell lysates from MNs with overexpression of Flag-tagged GFP or SMN1 in MNs from SMNi and SMA-1 groups (SMNi+GFP, SMA-1+GFP; SMNi+SMN1, SMA-1+SMN1) were analyzed by western blots using antibodies anti-Flag, -SMN1 and -NSE. Note, anti-SMN1 antibody reads two separate proteins: exogenously expressed Flag-SMN1 and endogenous residual SMN-FL produced by SMN2 gene. All the groups were collected and sampled under the same conditions. The cropped blots images are shown in the figure and the full-length blots are presented in Supplementary Fig. S3c. (**e**) Relative expression of total SMN-FL proteins measured by western blots in different MN groups in the 7^th^ week after differentiation (***p < 0.001; one-way ANOVA with post hoc test. N = 3 independent experiments). (**f**) Representative traces of induced APs upon current injections (from –40 pA to 100 pA with 10 pA step for 500 ms) in MNs from different groups. (**g**) Plot of AP frequency against injected current size for each group (***p < 0.001; two-way ANOVA. N = 18 ~ 20 neurons for each condition). All data shown represent mean ± SEM.

MNs from SMA transgenic mice^14^,^15^ and patients (this study) display hyperexcitability. To establish the relationship between the reduction of SMN-FL and the hyperexcitability, we performed two sets of experiments. First, we examined the AP activities on MNs differentiated from our established hESC lines with RNAi knockdown of SMN-FL (SMNi) or luciferase (Luc, as a control)^25^. qPCR and Western blot revealed a reduction of nearly 80% SMN-FL mRNA (Fig. 4a) and protein (Fig. 4b and Supplementary Fig. S3b). Using a similar recording regimen, we found that MNs from SMNi ESCs displayed an increased AP frequency and smaller rheobase as compared to the Luc group (Fig. 4c,g). This result suggests that SMN reduction results in MN hyperexcitability.

We then asked if SMN restoration corrects hyperexcitability in SMA MNs. By expressing flag-tagged SMN1 or GFP (control) in MNs that are differentiated from SMA-1 iPSCs and SMNi ESCs^25^, we observed a 5-fold increase in SMN-FL proteins in the SMN1 group as compared to the GFP groups (Fig. 4d,e, and Supplementary Fig. S3c). AP frequency was significantly reduced and the rheobase increased in the SMN1 groups upon current injections (Fig. 4f, g). Together, the gain and loss of function analyses suggest that the reduction of SMN-FL is responsible for hyperexcitability.

### SMA MNs exhibit increased Na^+^ channel currents

How SMN reduction results in MN hyperexcitability remains unknown. To reveal potential underlying mechanisms, we measured Na^+^-channel and K^+^-channel activities which are known to play crucial roles for AP patterns. By voltage-clamp, we found that 100% recorded neurons showed inward Na^+^ currents and outward K^+^ currents upon voltage jumps from −50 mV to +50 mV with a 10 mV step for 500 ms. To isolate K^+^ current (I_K_), we recorded neurons in the presence of tetrodotoxin (TTX; 1 μM) and CdCl_2_ (0.2 mM) in bath solution to block Na^+^- and Ca^2+^-channels, respectively (Supplementary Fig. S5a). The I_K_ density, calculated by plotting normalized I_K_ amplitude against membrane voltage, showed that SMA MNs displayed similar K^+^-channel activities as compared to control MNs (Supplementary Fig. S5b).

**Figure 5:**
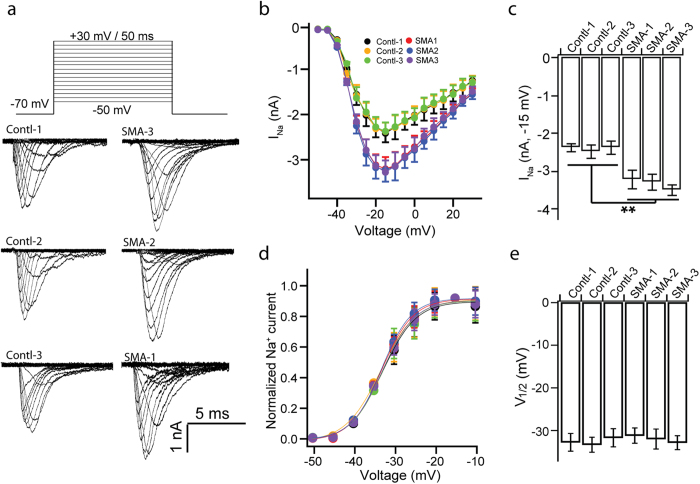
Na^+^ current properties in SMA MNs. (**a**) Representative sodium currents (I_Na_) elicited from a potential of −50 mV with a holding potential of −70 mV by 5 mV steps to potential of +30 mV in 50 ms duration in control and SMA MNs. Depolarization voltage steps of increasing amplitude were delivered every 3 s. (**b**) Current-voltage relations for the mean of peak currents evoked between −50 and +30 mV in 5 mV increments in control and SMA MNs. (**c**) Summary of the amplitude of peak I_Na_ at −15 mV for each group. (**d**) The peak amplitudes of Na^+^ currents were normalized to the peak of currents at −15 mV and plotted to depolarization voltage for each group. Data were fitted using equation 1 (lines) to calculate the potential of half-maximal activation (V_1/2_). (**e**) Quantification of V_1/2_ of sodium channel activation for each group. All data shown represent mean ± SEM. **p < 0.01; one-way ANOVA with post hoc test. N = 18 ~ 20 neurons for each group.

We next examined Na^+^ current (I_Na_) by giving a voltage jump for 50 ms from -50 mV to +30 mV with a 5 mV step in the presence of K^+^-channel blockers, tetraethylammonium (TEA; 12 μM) and 4-aminopyridine (4-AP; 1 mM) (Fig. 5a). At the same time, Ca^2+^-channels were blocked by CdCl_2_ (0.2 mM) in bath solution. The I_Na_ was plotted against membrane voltage to give the Na^+^-channel I-V curve (Fig. 5b). Interestingly, SMA MNs displayed significantly enhanced Na^+^ currents as compared to control MNs, while both SMA and control MNs exhibited maximum Na^+^ current at −15 mV (Fig. 5b). Nevertheless, the peak of Na^+^ current at −15 mV was significantly bigger in SMA MNs than in control MNs (Fig. 5c). To measure the voltage dependent activation of Na-channel, we normalized the peak current to the maximum peak current, and then plotted to the depolarizing voltage potentials (Fig. 5d). A Boltzmann function was used to fit the plot to tell the voltages of the half-activation (V_1/2_). We found that the V_1/2_ is similar for each group (Fig. 5e). Thus SMA MNs exhibit normal voltage dependent activation but enhanced Na^+^ currents upon stimulation.

### SMA MNs show faster recovery from Na^+^ channel inactivation

**Figure 6:**
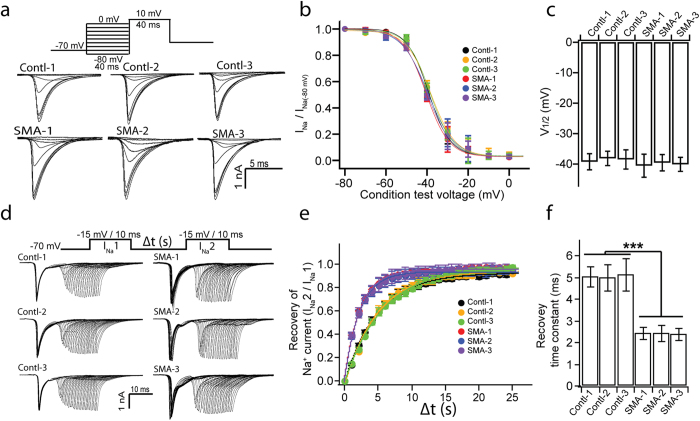
Inactivation and recovery of Na^+^ current in SMA MNs. (**a**) Representative current response evoked by step depolarization to 10 mV, applied every 3 s, from different condition potentials (from −80 mV to 0 mV for 40 ms in 10 mV increment) for each group. Upper panel illustrates the stimulation protocol. (**b**) The peak amplitude of I_Na_ were normalized to the peak of the first I_Na_ from condition potential of −80 mV (I_Na(−80 mV)_) and plotted vs condition voltage to construct the inactivation of sodium channels. Data were fitted using equation 1 (lines) to calculate potential for half-inactivation (V_1/2_, **c**) of sodium channels. (**c**) Quantification of V_1/2_ of inactivation for each group. (**d**) Representative I_Na_ traces upon to sequential depolarization with a series of inter-pulse interval (Δt) from different groups. Upper panel illustrates the stimulation protocol (Δt is from 1 ms to 25 ms in 1 ms increment) (**e**) Normalizing the peak amplitude of 2^nd^ Na^+^ current (I_Na_2) to 1^st^ Na^+^ current (I_Na_1), and then plotting the normalization vs Δt to reveal the time course of the recovery of Na^+^ currents. Data were fitted using equation 2 (lines) to calculate recovery time constant (**f**). (**f**) Summary of recovery time constant for each group. All data shown represent mean ± SEM. ***p < 0.001; one-way ANOVA with post hoc test. N = 18 ~ 20 neurons for each group.

Hyperexcitability may also be attributed to voltage dependent inactivation and recovery of Na^+^ channels. To investigate voltage dep-endent inactivation of Na^+^ channels, we recorded I_Na_ upon a voltage jump to +10 mV from a series of condition potentials (from −80 to 0 mV in 10 mV increment) (Fig. 6a). The amplitude of I_Na_ was normalized to the first response (at −80 mV) and plotted as a function of the condition potential (Fig. 6b). Similarly, when a Boltzmann function was used to reveal the voltages of the half-inactivation (V_1/2_), no difference was observed between SMA and non-SMA MNs (Fig. 6c), indicating that the voltage dependent inactivation of Na^+^ channel was not altered in SMA MNs.

To analyze the recovery of Na channel from inactivation, we used the two-pulse protocol. The protocol began with a condition depolarization from a holding potential of −70 to −15 mV for 10 ms, which inactivates the channels. This was followed by a second test pulse at the same depolarizing pulse value to elicit Na^+^ current. The time between the two pulses (Δt) was varied between 1 and 25 ms, in 1-ms increments, to determine the rate of recovery (Fig. 6d). We found that SMA MNs exhibited faster recovery from Na^+^ channel inactivation than control MNs (Fig. 6e, f).

**Figure 7:**
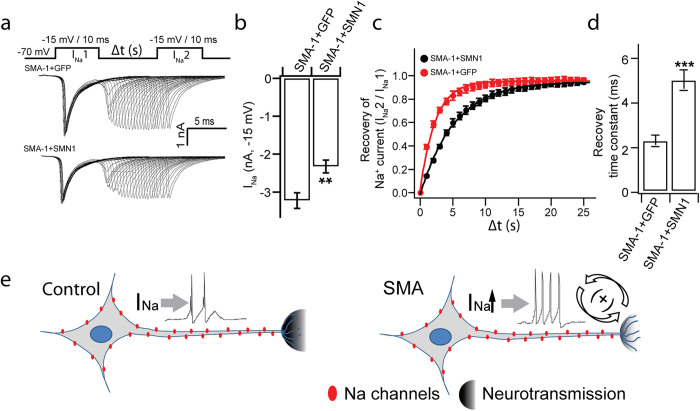
Na^+^ currents in SMA MNs following SMN expression. (**a**) Typical Na^+^ current traces from SMA-1 iPSC-derived MNs expressing Flag-GFP (SMA-1+GFP) or Flag-SMN1 (SMA-1+SMN1) upon sequential depolarization with a series of inter-pulse intervals. Upper panel illustrates the stimulation protocol (Δt is from 1 ms to 25 ms in 1 ms increment) (**b**) Quantification of the amplitude of the 1^st^ I_Na_ elicited at −15 mV depolarization. (**c**) Time course of the recovery of Na^+^ currents for each group. Data were collected and analyzed as described in Fig. 6e. (**d**) Summary of recovery time constant for each group (**e**) Model for hyperexcitability in SMA MNs. As compared to control MNs where there are normal AP activities and neurotransmission, the loss of SMN-FL in SMA MNs results into hyperexcitability due to increased Na^+^ channel activities and reduced neurotransmission, which perpetuate each other to maximize transmitter release upon stimulation. Such a “vicious” feedback results in failed neuromuscular transmission, contributing to severe symptoms at an early stage. All data shown represent mean ± SEM. **p < 0.01, ***p < 0.001; unpaired Student’s t-test. N = 18 neurons for each group from 2 independent experiments.

Since SMN reduction is responsible for hyperexcitability (Figs 3 and 4) and altered Na^+^-channel activities underlie the abnormal AP pattern, we hypothesize that restoration of SMN-FL corrects hyperexcitability by restoring Na^+^-current activities. Indeed, expression of SMN1 in SMA MNs (Fig. 4) decreased the peak amplitude of Na^+^-current (Fig. 7a,b). Similarly, the recovery of Na^+^-current in SMN1-expressing SMA MNs was significantly delayed as compared to the GFP group (Fig. 7c,d), close to the level seen in control MNs (Fig. 6f).

Together, our analysis revealed that SMA MNs display normal voltage dependent inactivation but faster recovery of Na^+^ channels and suggest that SMN regulates AP pattern likely by modifying Na^+^-current activities.

## Discussion

Human stem cells offer a model system to look at early events during pathogenesis. Using iPSCs that are reprogrammed from SMA patients and non-SMA individuals, we found that the differentiation of MNs is not altered by SMN mutation, at least during the first 7 weeks in culture when MNs are functional. Nevertheless, molecules that regulate transmitter and synaptic vesicles like ChAT and VAchT, are decreased in SMA MNs, suggesting potential effect on neuronal transmission. Interestingly, we have found that SMA MNs exhibited hyperexcitability with hyperpolarized threshold and larger AP amplitude, similar to what is observed in SMA transgenic mouse MNs. By restoring the SMN level in SMA MNs and by knocking down SMN in control MNs, we have now firmly established the causal-effect relationship between SMN mutation and MN hyperexcitability for the first time. Importantly, we have discovered enhanced Na^+^-channel activities in SMA MNs, including increased Na^+^ currents and faster recovery of Na^+^ channel activity from inactivation, which are corrected by SMN expression, suggesting it as an underlying cause of hyperexcitability. Together, we propose that reduction of *SMN* results in MN hyperexcitability and impaired neurotransmission, which exacerbates via a feedback loop, contributing to severe symptoms at an early stage of SMA (Fig. 7e).

SMA is generally regarded as a degenerative disease affecting primarily spinal MNs. Consistent with this, our present study using an SMA patient iPSC model shows that the generation of spinal MNs is not affected by SMN mutations, at least at an early stage (at 7 weeks after iPSC differentiation). This is somewhat different from recent reports using SMA iPSCs generated from similar sources of fibroblasts or using hESCs with SMN1 knockdown in which MNs were reduced at 6–8 weeks after PSC differentiation by 2–6 folds^22^,^23^,^25^. The reason behind the difference is not clear. One possibility is that we treated our cultures with compound E to prevent proliferation of neural progenitors and generation of new neurons from progenitors, whereas in previous studies new waves of neurons continue to differentiate from progenitors. Depending on the degree of progenitor proliferation and neuronal differentiation in SMA vs. non-SMA, the extent of reduction in MN proportion varies, which does not reflect MN degeneration. Our finding, to a large degree, is consistent with observations made in SMA transgenic animals which show very modest MN loss even at very late stages of the disease^10^,^11^,^12^,^13^. Therefore, we propose that MN loss is unlikely the major cause of SMA symptoms, at least at an early stage.

A critical question then is what underlies the severe symptom presentation and progressive nature of SMA. Our present finding suggests that altered MN membrane properties may be a critical element in the cascade of SMA pathogenesis. Increased input resistance and hyperpolarized threshold voltage allow SMA MNs to fire APs with less depolarized input, thus displaying hyperexcitability. Neuronal hyperexcitability is also observed in spinal cord MNs in transgenic SMA mice^14^,^15^ as well as in ALS^29^,^30^,^31^,^32^,^33^, indicating that MN excitability may be common in MN pathology. In the present study, we have established the causal-effect relationship between SMN reduction and MN hyperexcitbility in SMA by gain- and loss-of function analyses. Notably, GM00232 (one copy of SMN2) has a similar phenotype with GM03813 and GM09677 (2 copies of SMN2, Figs 3, 4, 5, 6), indicating that the dose-response relationship is not as clear based on the electrophysiological analysis on our cultured MNs.

How SMN mutation results in MN hyperexcitability remains unknown. Neuronal hyperexcitability may be attributed to decreased K^+^ currents and/or increased Na^+^ currents. In SMA MNs, we found larger Na^+^, but not K^+^, currents. These neurons display a higher AP amplitude and lower threshold voltage, suggesting greater availability of voltage-gated Na^+^ channels. Furthermore, we found faster recovery of Na^+^ currents, indicating persistent active Na^+^-channels during stimulation, thus enabling firing of more APs upon a given stimulation. Therefore, enhanced Na^+^-channel activities, including increased Na^+^ currents and their faster recovery, is responsible for the hyperexcitability of SMA MNs. By restoring the SMN level in the SMA MNs, we have further established that increased Na^+^ currents and faster recovery of Na channels mediate the effect of SMN reduction on MN excitability.

Besides hyperexcitability, MNs in SMA animal models show altered expression of synaptic proteins, suggesting impaired neurotransmission^11^,^34^,^35^,^36^. In SMA iPSC-derived MNs, VAchT and ChAT are reduced, suggesting potential impairment of synaptic vesicle release. Together, SMN reduction results in both hyperexcitability and impaired neurotransmission of MNs. Defective neurotransmission may enhance MN excitability to maximize synaptic vesicle release via a feedback mechanism. Therefore, we propose that hyperexcitability and reduced neurotransmission in SMA MNs may perpetuate each other to maximize transmitter release upon stimulation. Such a feedback system will eventually result in failed neuromuscular transmission because of further reduced availability of synaptic vesicles (Fig. 7e). Such a proposed mechanism explains the dichotomy between severe disease symptoms and mild neuronal degeneration in SMA. It also has important implications. It suggests that most MNs in SMA patients are only functionally impaired but not degenerated at early stages. This offers real opportunities to rescue MNs, and hence save patient life, by correcting genetic defects or functional deficits before MNs undergo degeneration.

## Methods

### Human pluripotent stem cells (PSCs)

Control fibroblasts (GM03814 and GM03815, Coriell Institute, Supplementary Table S1) and type-I SMA fibroblasts (GM03813, GM09677 and GM00232, Coriell Institute, Supplementary Table S1) were reprogrammed to iPSCs using retrovirus containing the Yamanaka factors, OCT4, SOX2, KLF-4 and c-MYC, as previous reported^28^. Pluripotency of the established iPSC lines was characterized by immunostaining for pluripotency markers and by teratoma formation in SCID mice. They were characterized for G banding karyotyping every 10 passages^37^. In addition, human WA09 ESC line (also known as H9, WiCell institute, NIH registry 0046) were used as an additional control. SMN1 knockdown (SMNi) and luciferase control (Luc) ESC lines were described before^25^. The PSCs were maintained on irradiated mouse embryonic fibroblasts as previously described^38^.

### MN Differentiation

PSCs were first differentiated to neuroepithelia (NE) in a neural medium consisting of DMEM/F12, N2 supplement, and non-essential amino acids in the presence of SB431542 (2 μM), DMH1 (2 μM), and CHIR99021 (3 μM, all from Stemgent) for 7 days. At d8, NE were treated with the addition of retinoic acid (RA, 0.1 μM) and purmorphamine (Pur, 0.5 μM) for 7 days for MN induction. At d14, MN progenitors were isolated and expanded as floating clusters in suspension in the same medium but without SB431542, DMH1 and CHIR99021 for an additional 7 days before plating on the laminin substrate for generating mature neurons. To generate synchronized post-mitotic neurons, the cultures were treated from d18-21 with compound E (0.1 μM, from Enzo) to block cell proliferation.

### Electrophysiology

Whole-cell voltage-clamp or current-clamp recordings were carried out at RT. The pipette solution consisted of (in mM) 145 K-gluconate, 0.1 CaCl_2_, 2 MgCl_2_, 1 EGTA, 2 Mg-ATP, 0.3 Na_3_-GTP, and 10 HEPES, pH 7.3 (290 mOsm). The bath solution consisted of (mM) 127 NaCl, 1.2 KH_2_PO_4_, 1.9 KCl, 26 NaHCO_3_, 2.2 CaCl_2_, 1.4 MgSO_4_, and 10 glucose, pH7.3 (295 mOsm). The bath solution was continuously bubbled with 95% O_2_/5% CO_2_ to maintain pH. To record Na^+^- currents, the pipette solution contained (in mM) 130 Cs methanesulfonate, 20 Tetraethyl-ammonium (TEA)-Cl, 2 EGTA, 1 MgCl_2_, 0.2 CaCl_2_, 2 Mg-ATP and 10 HEPES, pH 7.3 with CsOH (290 mOsm). For Na^+^- current recording, 2 mM 4-aminopyridine (4-AP) and 12 μM tetraethylammonium (TEA), and 0.2 mM CdCl_2_ were added to bath solution to block K^+^- and Ca^2+^- currents, respectively. For K^+^- current recording, 1 μM tetrodotoxin (TTX) and 0.2 mM CdCl_2_ were added to bath solution to block Na^+^- and Ca^2+^- current, respectively.

Neurons were visualized using an Olympus Optical (Tokyo, Japan) BX51WI microscope with differential interference contrast optics at 40× . Voltage-clamp and current-clamp recordings were obtained using a MultiClamp 700B amplifier (Molecular Devices). Signals were filtered at 4 kHz and sampled at 50 kHz using a Digidata 1322 A analog-to-digital converter (Molecular Devices). Only cells with series resistances of <15 M, with >80% of this resistance compensated, were analyzed. All data were analyzed using MiniAnalysis software (Synaptosoft), Clampfit (Molecular Devices), and Igor (Wavemetrics). All chemicals were purchased from Sigma-Aldrich.

Action potential amplitude was measured from the baseline to the peak of the voltage deflection. To hold the resting membrane potential (RMP) between −50 and −55 mV for measurements of AP amplitude, current injection was manually set at the beginning of the experiment and monitored/adjusted throughout. All neurons considered in this work for the current clamp experiments responded to current injection with sustained repetitive firing at a frequency that was comparatively linear as a function of the injected current. Na^+^ and K^+^ currents were elicited by voltage steps from a holding potential of −70 mV.

The input resistance (Fig. 2e) was measured by the slope of the linear portion of the current – voltage (I/V) relationship.

Activation (Fig. 5d) and inactivation (Fig. 6b) of voltage-dependent Na^+^- current were fitted with the Boltzmann equation.


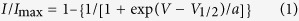


Where *V* is the potential of the given pulse, *V*_1/2_ is the potential for half-maximal activation and inactivation, and the *a* is the slope factor.

The recovery of sodium currents (Fig. 6e and Fig. 7c) were fitted with the following single exponential function equation


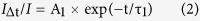


Where *I*_Δt_ is the amplitude of the current as a function of the time of recovery from inactivation, A_1_ is the relative percentage of current that recovery with the time constants τ_1_, t is the time, and *I* is the amplitude of the current related to the first depolarization.

### Alkaline phosphatase staining, immunocytochemistry and quantification

Alkaline phosphatase staining (Supplementary Fig. S1a) was performed using the Leukocyte Alkaline Phosphatase kit (Sigma).

The cultures of iPSCs and MNs were immunostained following standard procedures^39^. Briefly, cells were fixed in 4% paraformaldehyde for 15 min at 4 °C, washed with PBS, and incubated in a blocking buffer (10% donkey serum and 0.2% Triton X-100 in PBS) for 60 min at room temperature before being incubated in primary antibodies (Supplementary Table S2) overnight at 4 °C. Appropriate fluorescently conjugated secondary antibodies were used to reveal the binding of primary antibodies (1:1000, Jackson, West Grove, PA) and nuclei were stained with DAPI. Images were collected with a Nikon TE600 fluorescence microscope (Nikon Instruments, Melville, NY) or a Nikon C1 laser-scanning confocal microscope (Nikon, Tokyo, Japan).

To quantify the population of OLIG2^+^, MNX^+^, and ChAT^+^ cells among total cells (DAPI labeled) or neurons (TuJ1^+^), images were imported into ImageJ (NIH) for analysis. Cell counting was performed by a person blind to the experiment and replicated in different cell lines in three independent experiments.

### Western blotting

Cells were lysed using a buffer consisting of 62.5 mM Tris-HCl at pH 6.8, 20% glycerol, 2% SDS, 2 mM DTT, 100 μM PMSF and protease inhibitor cocktail. Lysates were resolved by SDS-PAGE and western blotting was carried out using horseradish peroxidase-conjugated IgG as a secondary antibody and the FlurChem HD2 imaging system (Proteinsimple, San Jose, CA) for detection.

### Infection of MNs

Lentiviral particles were generated by co-transfecting pLenti-Flag-GFP or pLenti-Flag-SMN1^25^ with two other packaging vectors encoding VSVG, and gag and pol, into HEK293T cells. The supernate was collected after 48–72 h, purified by filtration through a 0.45 μm filter and centrifuged at 70,000 g for 2 h to concentrate virus. Viral particles were re-suspended in phosphate-buffered saline (PBS), and were used to infect MNs at d28 after differentiation.

### RNA isolation and real-time PCR

Total RNA was isolated using the Trizol kit (Invitrogen, USA) according to manufacturer’s manual. 1 μg of total RNA from each sample was reversely transcribed into cDNA and subjected to real-time PCR using the Power SYBR Green kit (Applied Biosystems, UK). Primers for real-time PCR were listed in Supplementary Table S3.

### Teratoma formation

One million iPSCs were collected by dispase II (Life technologies-ThermoFisher Scientific, MA) and injected subcutaneously to dorsal flank of a SCID mouse. Two months after injection, tumors were dissected, weighted, and fixed with PBS containing 4% paraformaldehyde. Paraffin-embedded tissue was sliced and stained with hematoxylin and eosin (H&E).

### Karyotyping analysis and sequencing

Karyotyping of iPSC was performed by WiCell Institute (Madison, WI).

### Statistical analysis

All data were obtained from three independent experiments, unless otherwise indicated. For qPCR experiment, data were relative to GAPDH and normalized to H9 (Fig. 1I) or Luc (Fig. 4A) group. For western blotting experiment, data were relative to NSE and normalized to H9 (Fig. 1F, G, J), Luc (Fig. 4B), or SMNi +GFP (Fig. 4D) group. Statistical analyses were carried out by using Prism 6 software (Graphpad). No statistical methods were used to predetermine sample size. All data are presented as the mean ± SEM and significance was determined using the unpaired Student’s t test, one-way ANOVA, or two-way ANOVA (*p < 0.05, **p < 0.01, and ***p < 0.001), as appropriate.

## Additional Information

**How to cite this article**: Liu, H. *et al.* Spinal muscular atrophy patient-derived motor neurons exhibit hyperexcitability. *Sci. Rep.* **5**, 12189; doi: 10.1038/srep12189 (2015).

## Supplementary Material

Supplementary Information
