# Supplementary material for: Spinal muscular atrophy patient-derived motor neurons exhibit hyperexcitability
Source: Sci Rep. 2015 Jul 20;5:12189. doi: 10.1038/srep12189 (PMC4507262; doi:10.1038/srep12189)
Supplement: Supplementary Information [file srep12189-s1.pdf]

Supplementary information

Spinal muscular atrophy patient-derived motor neurons exhibit hyperexcitability

Huisheng Liu[1*, #], Jianfeng Lu[1*], Hong Chen[1], Zhongwei Du[1], Xue-Jun Li[3], and Su-Chun Zhang[1, 2 #]

[1]Waisman center, University of Wisconsin, Madison, WI, 53705, USA
[2]Department of Neuroscience and Department of Neurology, School of Medicine and Public Health, University of Wisconsin, Madison, WI 53705, USA
[3]Department of Neuroscience, University of Connecticut Health Center, Farmington, CT 06030, USA

* These authors contribute equally.
[#] Correspondence: Liu25@ Wisc.Edu or Zhang@Waisman.Wisc.Edu

Supplementary Figure S1
Supplementary Figure S2
Supplementary Figure S3
Supplementary Figure S4
Supplementary Figure S5
Supplementary Table S1
Supplementary Table S2
Supplementary Table S3

Supplementary Figure S1. Characterization of SMA and control iPSCs.
(a) Representative phase contrast images for fibroblasts and iPSCs as well as immunostaining images of iPSC clones generated from 2 controls and 3 type-I SMA patients.
(b) Karyotyping and G-banding analysis of iPSC lines.
(c) qPCR measurement for endogenous and exogenous pluripotent transcription factors (Relative to GAPDH and normalized to H9 group (Contl-1), n = 3 independent experiments). En, endogenous; Ex, exogenous; O, OCT4; S. SOX2; K, KLF-4; M, c-MYC; 4F / 3d, infection of 4 factors of O, S, K and M (4F) for 3 days.
(d) H&E staining of sections of teratomas from iPSCs showing cartilage (mesoderm), gut epithelia (endoderm) and epiderm (ectoderm) like structures.
All data shown represent mean ± SEM.

Supplementary Figure S2. SMA iPSCs generate MNs as efficiently as control PSCs.
(a) Representative images of MNPs, identified by OLIG2, from control and SMA NS at d14 after differentiation.
(b) Representative $MNX1^{+}$ MNs generated from control and SMA iPSCs 48-hour after plating at d21 after differentiation.
(c) Representative $ChAT^{+}$ MNs at d49 after differentiation.

Supplementary Figure S3. Full-length blots for key data in the main figures.
(a - c) Full length of western-blot images of Figure 1e and j (a), Figure 4b (b) and Figure 4d (c).

Supplementary Figure S4. Investigating electrophysiological activities in MNs differentiated from control and SMA iPSCs.
Representative images of recorded neurons at the 7th week after differentiation from normal control and SMA iPSCs. Neurobiotin was injected to neurons during recording and used to identify the type of cells after recording

Supplementary Figure S5. SMA MNs display normal potassium channel activities.
(a) Representative potassium currents (I_k) elicited from -50 mV with a holding potential of – 70 mV by 10 mV steps to potential of +50 with 500 ms duration in control and SMA MNs. 1 µM TTX and 0.2 mM Cd^{2+} were added in bath solution to block Na^{+} and Ca^{2+} currents, respectively. Depolarization voltage steps of increasing amplitudes were delivered every 3 s.
(b) Current-voltage relations for the normalized peak currents to membrane capacitance, evoked between -50 and +50 mV in 10 mV increments in normal control and SMA MNs. Inset: enlargement of the last two data points. For each condition, we recorded from 18-

20 cells from a total of six coverslips, where two coverslips were obtained from each of three independent experiments. All data shown represent mean ± SEM.

Supplementary Table S1. Stem cell lines and replication, related to Human Pluripotent Stem Cells (PSCs)

Group	Cat. ID	Diagnosis	Gender	Genetic background	Age at biopsy	clones	replications
Contl-1	WA09 (known as H9)	Normal	N/A	N/A	N/A	1	3
Contl-2	GM03814	Carrier	Female	2 copies of *SMN2*, heterozygous Δ*SMN7*/8 of *SMN1*.	Adult	1	3
Contl-3	GM03815	Carrier	Male	2 copies of *SMN2*, heterozygous Δ*SMN7*/8 of *SMN1*.	Adult	1	3
SMA-1	GM03813	Type-1	Male	2 copies of *SMN2*, homozygous Δ*SMN7*/8 of *SMN1*.	3y	1	3
SMA-2	GM09677	Type-1	Male	2 copies of *SMN2*, homozygous Δ*SMN7*/8 of *SMN1*.	2y	1	3
SMA-3	GM00232	Type-1	Male	1 copies of *SMN2*, homozygous Δ*SMN7*/8 of *SMN1*.	7m	1	3

Supplementary Table S2. Primary antibodies, related to Alkaline Phosphatase Staining, Immunocytochemistry and Quantification

Antibody	Isotype	Dilution	Source
ChAT	Goat IgG	1:300	Chemicon &Millipore
Flag	Mouse IgG	1:1000	Sigma
SMN	Rabbit IgG	1:500	Santa Cruz
MNX1/H9	Mouse IgG	1:50	DSHB
NONAG	Rabbit IgG	1:500	R&D
NSE	Rabbit IgG	1:1000	Abcam
OCT4	Mouse IgG	1:500	Chemicon &Millipore
OLIG2	Rabbit IgG	1:500	Chemicon &Millipore
SEEA4	Mouse IgG	1:1000	DSHB
SOX2	Goat IgG	1:1000	R&D
TRA-1-60	Mouse IgG	1: 50	Chemicon & Millipore
TuJ1	Mouse IgG	1:200	Chemicon & Millipore
VAChT	Rabbit IgG	1:1000	Sigma

Supplementary Table S3. Primers for real-time PCR and DNA PCR

Name	Forward Primer Sequence	Reverse Primer Sequence
Real-time PCR primer		
EXO-OCT4	5’GGGTGGACCATCCTCTAGAC3’	5’CCAGGTCCGAGGATCAAC3’
EXO-SOX2	5’GGGTGGACCATCCTCTAGAC3’	5’GGGCTGTTTTTCTGGTTG3’
EXO-KLF-4	5’GGGTGGACCATCCTCTAGAC3’	5’GGAAGTCGCTTCATGTGG3’
EXO-c-MYC	5’GGGTGGACCATCCTCTAGAC3’	5’CCTCGTCGCAGTAGAAATAC3’
EN-OCT4	5’AGTTTGTGCCAGGGTTTTTG3’	5’ACTTCACCTTCCCTCCAACC3’
EN-SOX2	5’CAAAAATGGCCATGCAGGTT3’	5’AGTTGGGATCGAACAAAAGCTATT3’
EN-KLF-4	5’AGCCTAAATGATGGTGCTTGGT3’	5’TTGAAAACTTTGGCTTCCTTGTT3’
EN-c-MYC	5’CGGGCGGGCACTTTG3’	5’GGAGAGTCGCGTCCTTGCT3’
SMN-FL	5’ATGTTAATTTCATGGTACATG3’	5’GGAATGTGAGCACCTTCCTTC3’
GAPDH	5’GAAGGTGAAGGTCGGAGTC 3’	5’GAAGATGGTGATGGGATTTC3’
DNA PCR primer (for Dde1 experiment)		
SMN	5’CAAGCCCAAATCTGCTCCAT 3’	5’TACAATGAACAGCCATGTCC3’

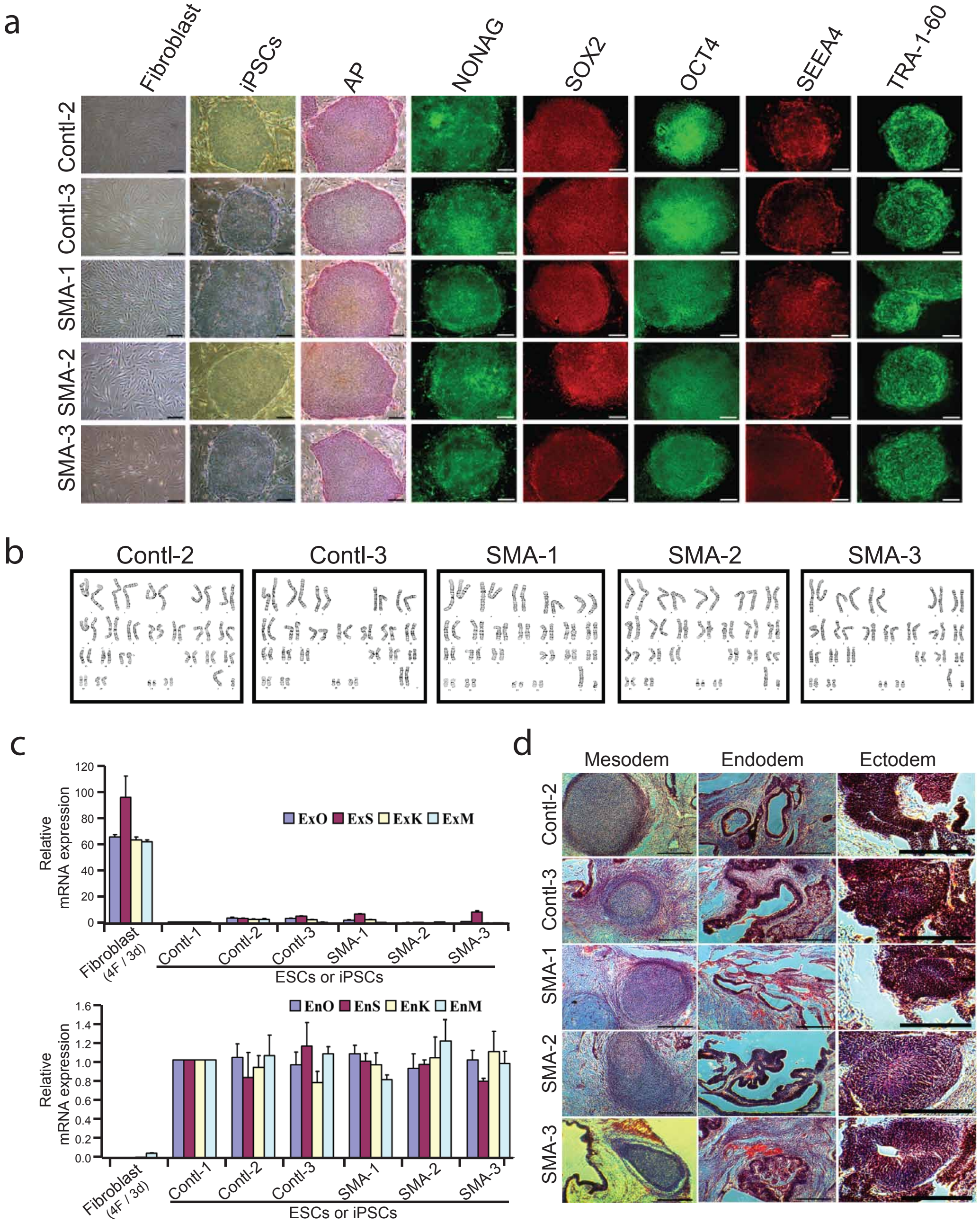

Supplementary Figure S1

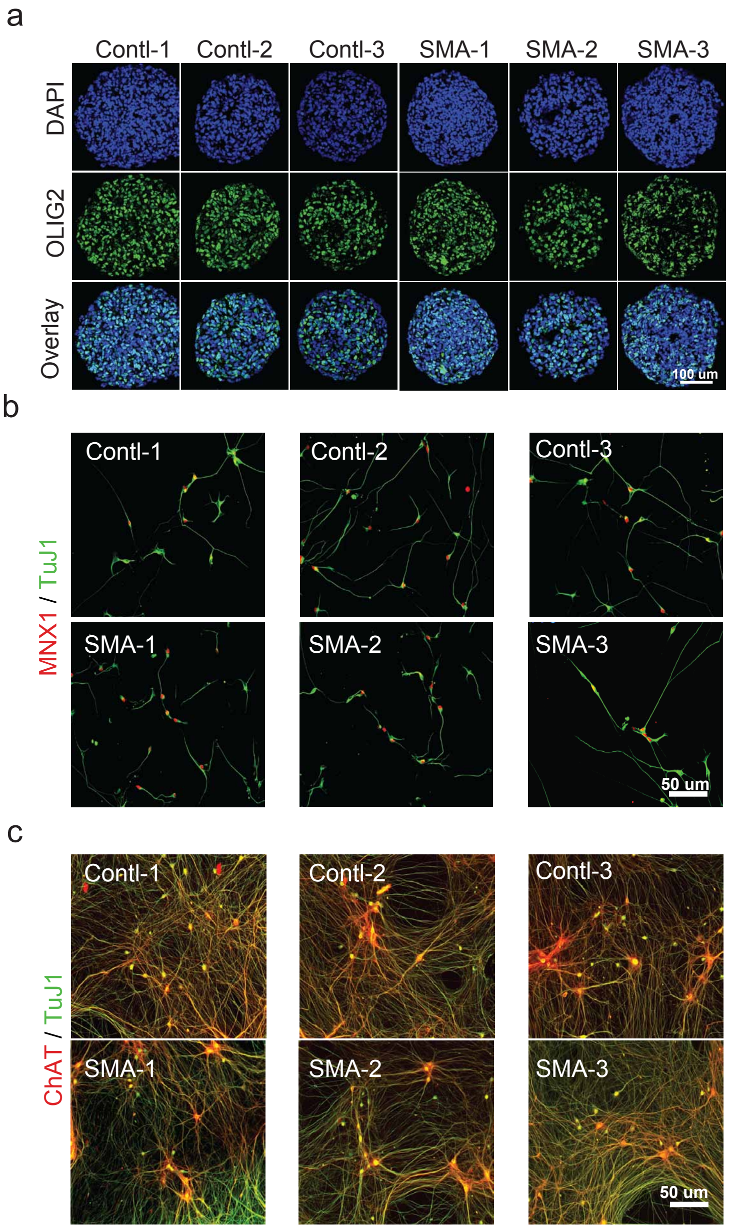

Supplementary Figure S2

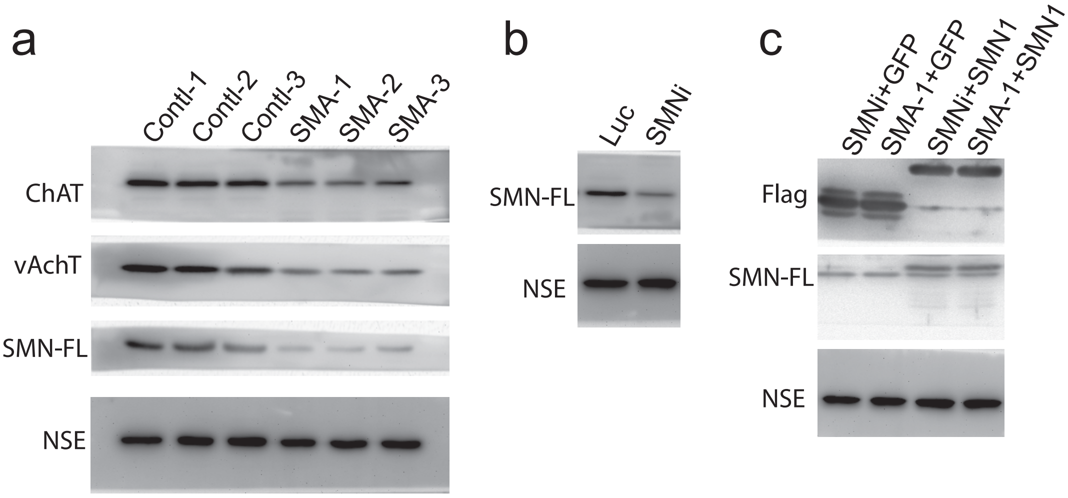

Supplementary Figure S3

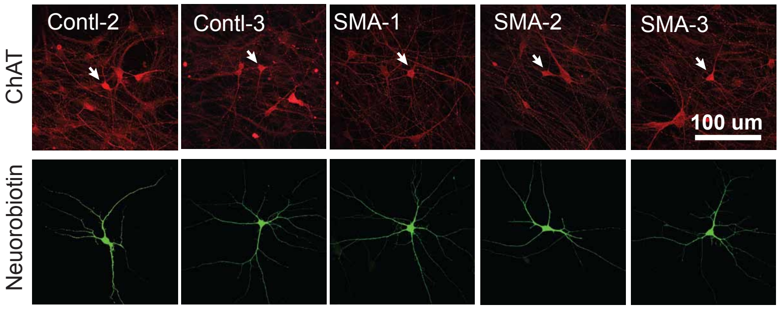

Supplementary Figure S4

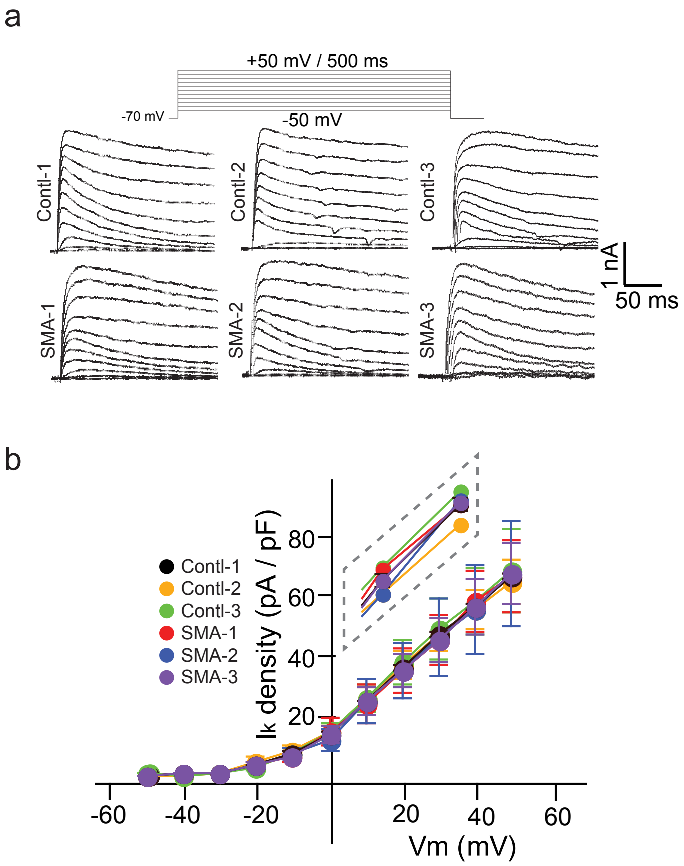

Supplementary Figure S5
